# Editorial: Dynamic functional connectivity in neuropsychiatric disorders: Methods and applications, volume II

**DOI:** 10.3389/fnins.2022.1092718

**Published:** 2022-11-28

**Authors:** Xiaoya Fu, Feng Liu, Zaixu Cui, Yuqi Cheng, Zhifen Liu, Wenbin Guo

**Affiliations:** ^1^Department of Psychiatry, National Clinical Research Center for Mental Disorders, The Second Xiangya Hospital of Central South University, Changsha, China; ^2^Department of Radiology, Tianjin Medical University General Hospital, Tianjin, China; ^3^Chinese Institute for Brain Research, Beijing, China; ^4^Department of Psychiatry, The First Affiliated Hospital of Kunming Medical University, Kunming, China; ^5^Yunnan Clinical Research Center for Mental Disorders, The First Affiliated Hospital of Kunming Medical University, Kunming, China; ^6^Department of Psychiatry, First Hospital of Shanxi Medical University, Taiyuan, China; ^7^Shanxi Key Laboratory of Artificial Intelligence Assisted Diagnosis and Treatment for Mental Disorder, First Hospital of Shanxi Medical University, Taiyuan, China; ^8^Department of Psychiatry, The Third People's Hospital of Foshan, Foshan, China

**Keywords:** structural MRI, functional MRI, cognitive deficits, depression, anxiety

In 2010, the Global Burden of Disease Study (GBD) reported that the disease burden of neuropsychiatric disorders accounted for 10.4% of global disability-adjusted life years (DALYs). Psychiatric disorders, the fifth leading cause of DALYs, accounted for 7.4% of global DALYs, while neurological disorders accounted for 3% of global DALYs (Whiteford et al., 2015). As the population grows and ages, the global burden of neuropsychiatric diseases continues to increase, which makes neuropsychiatric disorders a prominent health issue.

Patients with neuropsychiatric disorders show multiple problems regarding to emotion, cognition, behavior, and physical symptoms. The neuropathological alterations underlying these somatic discomforts and functional deficits have been one of the critical concerns of researchers. Magnetic resonance imaging (MRI), a safe and non-invasive way to detect changes in brain structure and function, has been one of the most important tools for studying neuropathological changes in neuropsychiatric diseases. Since the discovery of resting-state functional connectivity (FC) by Biswal et al. (1995) and the subsequent introduction of the default mode network (Biswal et al., 1995; Raichle et al., 2001), functional MRI has greatly broadened and deepened our understanding of brain function over the past two decades. However, the focus shifts toward temporal fluctuations in blood-oxygen-level-dependent (BOLD) FC in the brain, considering the rapidly changing neural activity of the brain. In addition to focusing on the dynamics of FC, the temporal fluctuations of the amplitude of low-frequency fluctuation (ALFF), regional homogeneity (ReHo), and other metrics commonly used in resting-state functional MRI have also been explored for investigating the neuropathological alterations of neuropsychiatric disorders.

This issue is a continuum of our previous topic (Fu et al., 2020). In this Research Topic, we received several articles applying neuroimaging tools to reveal the dynamic function of the brain and articles focusing on neuropsychiatric disorders with other imaging analyses. In the end, a total of 18 papers are included in this Research Topic. We found that the concerns of many studies shifted from a particular disorder to more specific symptoms. Given the heterogeneous manifestations of neuropsychiatric disorders, this symptom delineation might be more conducive to the exploration of the neuropathological mechanisms underlying specific symptoms. In addition, many studies explored the correlation of imaging findings with clinical symptoms and the robustness of differences between groups. These attempts might have a positive influence on the clinical application of these imaging tools.

Of these 18 articles, only 2 articles examined neurological disorders, and the other 16 articles focused on psychiatric disorders. Major depressive disorders (MDD) were the most discussed disorders. Suicide, non-suicidal self-injury, childhood trauma, and medication efficacy were the main focus of these MDD studies.

MDD was responsible for the highest proportion of neuropsychiatric disorder DALYs (Whiteford et al., 2015). Both suicide and non-suicidal self-injury are strongly associated with MDD. Li W. et al. and He et al. examined the neural basis of suicide ideation (SI) of patients of MDD by using different imaging analyses in various brain regions. Li W. et al. used a dynamic FC to study a critical brain region of a default mode network, the PCC, which has been reported to be associated with suicidal ideation in depressed patients in structural and functional MRI studies (Schmaal et al., 2020). He et al. focused on the neuropathological alterations of cingulo-opercular network in MDD patients with SI by using structural and functional neuroimaging. Besides SI, non-suicidal self-injury (NSSI) is also a symptom worthy of note, especially in adolescent MDD. Both NSSI and SI exhibited robust relationships to attempted suicide (Klonsky et al., 2013). Liu H. et al. used an electrophysiological approach to explore the difference in P300 in adolescent MDD patients with NSSI. Yang C. et al. concentrated on suicide attempts (SA) in patients with MDD, and they found that elevated activity in the cingulum functioning may be related to SA.

The associations between childhood trauma and multiple psychiatric disorders have been reported in many previous studies (Varese et al., 2012; McKay et al., 2021). Childhood trauma is a significant predictor of depression severity (Hopfinger et al., 2016). Chen et al. investigated the functional MRI data of female MDD patients with and without childhood trauma and healthy controls. But no ALFF difference was found between MDD patients with childhood trauma and those without. Luo et al. also showed an interest in the impact of childhood trauma on the brain function of patients with MDD. They used multiple dynamic functional MRI indices throughout the whole brain, and examined the aberrant temporal fluctuations of brain function from various perspectives.

As a non-invasive examination, MRI is also very promising in predicting drug efficacy. Two articles in this special issue examined the possibility that MRI is used to predict drug efficacy for two kinds of medical intervention, respectively. Zhang A. et al. separated patients with MDD into a responsive group and a non-responsive group based on the reduction rate of the scores of Hamilton Depression Rating Scale (HAMD-17) after 2-week SSRI treatment and found that there were differences in ReHo in the right parahippocampal gyrus and the middle temporal gyrus between groups. Compared to the study of Zhang A. et al., Zhang F. et al. focused on a novel antidepressant, ketamine, an N-methyl-D-aspartate (NMDA) antagonist. The responders in this study were defined as having an improvement in the Montgomery-Asberg Scale (MADRS) scores ≥ 50% after six intravenous injections of ketamine over 12 days. Zhang F. et al. found that responders had lower values of degree centrality in the right middle frontal gyrus (MFG) and stronger FC between the MFG and the right supplementary motor area (SMA) than non-responders.

Besides studies concerning MDD, there are also studies in this special issue that focus on dynamic brain function changes in other psychiatric disorders. Kong et al. investigated network homogeneity (NH) of the default mode network in 57 first-diagnosis drug-naïve schizophrenic patients and 50 healthy controls. Fateh et al. evaluated the dynamic FC within six subdivisions of the insula to investigate whether the dysregulated dynamic FC in insula was related to social dysfunction in patients with attention deficit hyperactivity disorder (ADHD). Liu D. et al. examined both static and dynamic functional brain network in adults with problematic smartphone use. It is important to note that although both the International Classification of Diseases (11 Edition, ICD-11) and Diagnostic and Statistical Manual of Mental Disorders (Fifth Edition, DSM-5) now include a diagnosis of gaming disorder (Gaming Disorder in ICD-11 and Internet Gaming Disorder in DSM-5), problematic smartphone use is not currently a disease. Liu D. et al. found no group difference between participants with and without problematic smartphone use, however the severity of problematic smartphone use was correlated with FC strength as well as temporal variability. Yang, Li, et al. conducted the only comparative study on psychiatric disorders in this special issue. They recruited patients with schizophrenia (SZ), major depressive disorder (MDD) and bipolar disorder (BD) and healthy controls to examine the common and specific neuroanatomical characteristics.

There are three articles in this special issue involved in other diseases rather than psychiatric disorders. Yang, Zhao, et al., and Su et al. all concerned about systemic lupus erythematosus (SLE), an autoimmune disease sometimes involving the nervous system, called neuropsychiatric systemic lupus (NPSLE). The study of Su et al. demonstrated that patients with NPSLE showed atrophic subcortical gray matter and functional alterations in the default mode network, salience network, sensorimotor network, and cerebellum. Yang, Zhao, et al. explored the static and dynamic ALFF in SLE patients with cognitive impairment (CI). Patients with SLE showed altered dynamic ALFF no matter with or without CI, and SLE patients with CI also had changed static ALFF. Nevertheless, compared to SLE patients without CI, those with cognitive impairment only showed higher static ALFF in the right parahippocampal gyrus, but no difference in dynamic ALFF was found between these two groups. Wang, Wang, et al. used FC to discriminate between migraineurs and tension-type headache, which share many similarities in clinical practice.

Notably, three articles in this special issue introduced novel analysis methods for neuropsychiatric disorders. These three articles all proposed improvements and reflections on the application of image analysis to disease diagnosis. Wang, Fu, et al. used the random support vector machine (SVM) cluster, a machine-learning framework, to extract an optimized random SVM cluster that performs well in classifying patients with autism spectrum disorder (ASD) and healthy controls. Song et al. proposed a novel brain-network-constrained multi-view sparse canonical correlation analysis (BN-MSCCA), which combined the structural and functional MRI data as well as diagnosis information to explore the schizophrenia-related biomarkers. Both articles introduced multiple or multimodal features when testing the diagnostic model. Li Y. et al. combined uncertain brain networks and a novel discriminative feature selection method based on a statistical index (dfsSI) to optimize the time consumption, computational cost and the classification accuracy.

Overall, the articles in this special issue applied various imaging analysis methods, with a particular focus on the dynamic changes in brain function. These articles explored the underlying neural basis of neuropsychiatric disorders and also presented several novel approaches. It is hoped that clinicians and researchers will benefit from them.

## Author contributions

All authors listed have made a substantial, direct, and intellectual contribution to the work and approved it for publication.

## Funding

This study was supported by grants from the National Natural Science Foundation of China (Grant No. 82171508), Natural Science Foundation of Hunan (Grant No. 2020JJ4784), Science and Technology Program of Hunan Province (Grant No. 2020SK53413), and Natural Science Foundation of Tianjin (Grant No. 18JCQNJC10900).

## Conflict of interest

The authors declare that the research was conducted in the absence of any commercial or financial relationships that could be construed as a potential conflict of interest.

## Publisher's note

All claims expressed in this article are solely those of the authors and do not necessarily represent those of their affiliated organizations, or those of the publisher, the editors and the reviewers. Any product that may be evaluated in this article, or claim that may be made by its manufacturer, is not guaranteed or endorsed by the publisher.
